# Long Noncoding RNA HCG9 Promotes Osteosarcoma Progression through RAD51 by Acting as a ceRNA of miR-34b-3p

**DOI:** 10.1155/2021/9978882

**Published:** 2021-08-18

**Authors:** Lu Wang, ShuangQing Li, Lin Qi, Lin Ling

**Affiliations:** Department of Orthopedics, The Second Xiangya Hospital, Central South University, Changsha, Hunan 410011, China

## Abstract

**Background:**

Long noncoding RNAs (lncRNAs) have critical regulatory functions in biological and pathological activities during osteosarcoma progression. It is important to elucidate the expression pattern and reveal the underlying mechanisms of the newly identified lncRNAs.

**Methods:**

Herein, we screened the differentially expressed lncRNAs in osteosarcoma tumors and cell lines using lncRNA microarray. The candidate lncRNA was further verified by qRT-PCR, and the association of gene expression with clinicopathological features was evaluated by Kyoto Encyclopedia of Genes and Genomes (KEGG) pathway analysis. The targeting miRNA was identified using starBase analysis, and the competing endogenous RNA (ceRNA) network was established by STRING. Overexpression and silence of RNA were detected by qRT-PCR. Osteosarcoma cell proliferation was measured with CCK-8 assay, and the migration and invasion were evaluated with Transwell assay. Colony formation assay was observed. Flow cytometry evaluated the cell cycle. Western blot was performed to detect the mitotic markers and apoptosis-related proteins. A nude mouse tumor formation experiment was used to evaluate osteosarcoma progression *in vivo*. Cooverexpressing miR-34b-3p with RAD51 reversed the miR-34b-3p-induced changes in proliferation, the cell cycle, the expression of H2A.X, and that of apoptosis-related proteins.

**Results:**

HCG9 was identified as osteosarcoma-associated lncRNA. Osteosarcoma tissues and cell lines expressed higher levels of HCG9 as compared to normal tissues and osteoblasts, and high expression of HCG9 was further proved to be related to metastasis and the grade of osteosarcoma in clinical cases. Knockdown of HCG9 inhibited the proliferation, migration, and invasion of osteosarcoma cells. miR-34b-3p was identified as the target of HCG9, and RAD51 acted as a potential target of miR-34b-3p. Cooverexpressing miR-34b-3p with HCG9 partially suppressed the HCG9-stimulated proliferation, migration, and invasion of osteosarcoma cells *in vitro* and delayed the tumor progression *in vivo*.

**Conclusion:**

We discovered that lncRNA HCG9 promoted the proliferation of osteosarcoma cells via suppressing miR-34b-3p. Our study provides novel biomarkers and potential therapeutic targets for osteosarcoma treatment.

## 1. Introduction

Osteosarcoma is the most common primary bone malignancy that occurs predominantly in adolescents [1]. Despite the advancement in diagnosis, chemotherapy, and surgical techniques, the prognosis of osteosarcoma remains poor. The long-term survival rate of localized osteosarcoma is 77%, while this disease becomes more lethal when metastasis occurs, with the long-term survival rate dropping to 26% according to the American Cancer Society. Although the underlying molecular mechanism of osteosarcoma remains unclear, extensive efforts have been expanded to explore more targeted and localized treatment for osteosarcoma [2]. With the increasing knowledge of molecular pathogenesis, gene therapy has emerged as a controlled, targeted, and specific treatment for osteosarcoma [3, 4].

Over 90% of the human genome does not encode proteins, recognized as “noncoding RNAs” (ncRNAs) [5]. Long noncoding RNAs (lncRNAs) are a class of ncRNAs that contain over 200 nucleotides. Increasing pieces of evidence have shown that dysregulated lncRNA gene expression is associated with tumor progression [6–8]. The first study on lncRNA in osteosarcoma was performed by Li et al., who identified over 400 upregulated and 798 downregulated lncRNAs in osteosarcoma by lncRNA expression microarray, providing valuable potential biomarkers for osteosarcoma [9]. Gao and Lian reported that lncRNA MALAT1 was an independent prognostic factor of osteosarcoma [10], and another lncRNA, H19, was also proved to be associated with osteosarcoma progression [11]. HCG9 is also known as the human leukocyte antigen complex group 9 gene, and studies have reported that HCG9 was related to lung squamous cell carcinoma [12] and nasopharyngeal carcinoma [13]. However, the role of HCG9 in osteosarcoma remains unknown.

One of the major functions of lncRNAs is acting as competing endogenous RNAs (ceRNAs) to regulate lncRNA/miRNA/mRNA crosstalk [14]. The ceRNA hypothesis was proposed by Salmena et al. [15]. They hypothesized that lncRNAs are complete for the miRNA binding sites via partial complementarity, which leads to a decreased miRNA activity for mRNA regulation. Since then, many researchers have validated this theory [16]. In osteosarcoma specifically, Pan et al. reported that lncRNA FBXL19-AS1 sponged miR-346 to regulate osteosarcoma cell proliferation [17]. Zheng et al. connected the lncRNA SNHG3/miR-151a-3p/RAB22A functioning axis to regulate osteosarcoma invasion and migration [18]. However, many potential lncRNA/miRNA regulations have not yet to be identified.

In this study, we performed lncRNA microarray analysis and identified HCG9 as one of the differentially expressed lncRNAs in osteosarcoma tissues. To our knowledge, this is the first report to associate HCG9 with osteosarcoma progression. We established a ceRNA network, and the targeting miRNA miR-34b-3p was pinpointed. The interaction between HCG9 and miR-34b-3p was validated with bioinformatic analysis and molecular biological analysis, and we also reported that RAD51 was the potential downstream mRNA targeted by miR-334b-3p. The impact of HCG9 knockdown and overexpression on osteosarcoma cells *in vitro* and tumor progression *in vivo* was evaluated. Our results provided novel biomarkers of osteosarcoma and revealed the theoretical basis of the underlying mechanisms.

## 2. Materials and Methods

### 2.1. lncRNA Microarray Chip and Bioinformatic Analysis

lncRNA gene expressions in osteosarcoma tissues and paracarcinoma tissues were analyzed. The GSE21257 dataset from the GEO database (https://www.ncbi.nlm.nih.gov/gds/) was used as the reference. Univariate Cox regression analysis was used to identify the differentially expressed lncRNAs. A minimum *X*-fold change in gene expressions between tumor tissue and normal tissue with *P* < 0.05 was screened. The Cancer Cell Line Encyclopedia database was used as the reference to validate HCG9 expression in cancer cells.

The osteosarcoma survival information was used to generate Kaplan-Meier separate survival analysis and log-rank tests for the correlation between HCG9 expression and survival rates. Kyoto Encyclopedia of Genes and Genomes (KEGG) pathway analysis was conducted to establish the association between HCG9 and clinicopathological features of osteosarcoma.

The ceRNA network was generated by assessing the interaction among proteins with STRING (2017 release) in the standard combined score > 0.4. The visualization of the protein-protein interaction (PPI) network was obtained with Cytoscape software (version 3.6.1). Molecular Complex Detection (MCODE, version 1.31) was adopted to identify the significant modules and the top-ranked genes in the PPI network.

### 2.2. Tissue Collection

A total number of 47 patients who received tumor removal surgeries from the Second Xiangya Hospital were recruited. 15 pairs of osteosarcoma and paracarcinoma tissues were collected from the patients who consented to all the investigations in this study. Patients were all informed and signed written consent before surgery. All procedures and tissue collection protocols were approved by the Institutional Review Board of the Second Xiangya Hospital. Tissue specimens were stored in liquid nitrogen or fixed in 10% formalin before the execution of experiments.

### 2.3. Cell Culture and Transfection

Osteoblast cell line hFOB1.19 and osteosarcoma cell lines U2OS, MG-63, and MNNG/HOS were all purchased from ATCC (Manassas, VA). Cells were cultured in Dulbecco's Modified Eagle's Medium (DMEM) (Gibco) containing 10% fetal bovine serum in a 5% CO_2_ atmosphere at 37°C. For cell transfection, HCG9 overexpressing plasmid, HCG9 small interfering RNAs (siRNAs), and negative controls were purchased from Thermo Fisher Scientific Inc. miR-34b-5p mimics and negative controls were purchased from Qiagen. The transfection was performed using Lipofectamine 2000 (Thermo Fisher Scientific, Waltham, MA) according to the manufacturer's protocol. Cells were harvested for characterization at 48 hours posttransfection.

### 2.4. Transwell Assay

Cell migration and invasion were both assessed with the Transwell system. For migration assay, 2 × 10^4^ cells were suspended in 100 *μ*L serum-free DMEM and seeded in the upper chambers of an 8 *μ*m pore size Transwell plate (Corning, USA). For invasion assay, the upper chamber of an 8 *μ*m pore size Transwell plate was pre-coated with the Matrigel™ Matrix (BD Biosciences, CA, USA), and 2 × 10^4^ cells were seeded and cultured in serum-free DMEM.

For both assays, a total volume of 500 *μ*L DMEM containing 10% fetal bovine serum (Gibco) was added to the lower chambers. After 24 h in culture, the migrated and invaded cells at the bottom surface were fixed with 4% paraformaldehyde and stained with 1% crystal violet. The cells were observed with a Zeiss upright microscope. The staining was washed using 33% acetic acid, and the absorption at 590 nm was analyzed with a microplate reader (Thermo Fisher Scientific).

### 2.5. Cell Counting Kit-8 (CCK-8) Assay

Cell proliferation was evaluated with the CCK-8 assay (Dojindo, Japan). Wild-type control and transfected cells were harvested and seeded into a 96-well plate. Three technical repeats were performed for each group. After cell adherence and 24 h of culture, 10 *μ*L CCK-8 solution was added to each well and incubated at 37°C for 4 h. Cell proliferation was evaluated with a microplate reader (Bio-Rad Laboratories, Hercules, CA) at 450 nm.

### 2.6. Colony Formation

Wild-type and transfected cells were seeded in a 6-well plate with a density of 800 cells/well. After 10 days in culture, the cells were fixed with 4% paraformaldehyde and stained with 1% crystal violet. The staining was washed using 33% acetic acid, and the absorption at 590 nm was analyzed with a microplate reader (Thermo Fisher Scientific).

### 2.7. Quantitative Real-Time Polymerase Chain Reaction (qRT-PCR)

Total RNA was isolated from osteosarcoma tumor tissues, paracarcinoma soft tissues, and cell lines using a TRIzol® reagent (Invitrogen, CA, USA). cDNAs were synthesized using a PrimeScript™ RT reagent kit (ComWin Biotech, Beijing, China). Primers of HCG9 and miR-34b-5p were obtained from Thermo Fisher Scientific. The primer sequences are shown in Table 1. PCR was performed with the SYBR Green Master Mix (ComWin Biotech, Beijing, China) using a fluorescent quantitative PCR system (Thermo Fisher Scientific, USA). The relative expression levels were calculated using the 2^-*ΔΔ*Ct^ method, and GAPDH and U6 were used as the internal reference for mRNAs and miRNAs, respectively.

### 2.8. Western Blot

Cells or tissues were lysed in a RIPA lysis buffer (Beyotime Biotechnology, Shanghai, China) for 10 min on ice. The protein concentrations were determined using a BCA Protein Assay Kit. An equal amount of protein was loaded and separated using SDS-polyacrylamide gel electrophoresis. The proteins were then transferred to a nitrocellulose membrane. Membranes were blocked in a 1x PBS buffer containing 5% nonfat milk and 0.1% Tween-20 (PBST) for 1 h at room temperature, then subsequently stained with primary antibodies overnight at 4°C, including *β*-actin (mouse, 1 : 5000; cat# 66009-1-Ig; Proteintech, USA), H2A.X (mouse, 1 : 1000; cat# ab26350; Abcam, UK), RAD51 (rabbit, 1 : 1000; cat# ab133534; Abcam, UK), p53 (mouse, 5 *μ*g/mL; cat# ab26; Abcam, UK), Bcl-2 (rabbit, 1 : 1000; cat# 12789-1-AP; Proteintech, USA), Bax (rabbit, 1 : 1000; cat# 50599-2-Ig; Proteintech, USA), and Caspase-3 (rabbit, 1 : 1000; cat# 19677-1-AP; Proteintech, USA). After washing 3 times with PBST, the membranes were incubated with horseradish peroxidase-conjugated goat anti-rabbit (1 : 6000; cat# SA00001-2; Proteintech, USA) or goat anti-mouse antibodies (1 : 5000; cat# SA00001-1; Proteintech, USA) for 1.5 h at room temperature. After 1 min incubation with enhanced chemiluminescence (SuperECL Plus, Advansta, USA), the signal intensity was detected by X-ray.

### 2.9. Cell Cycle Analysis

Cells in different transfection groups were collected and washed with PBS. After fixation with ice-cold 70% (*v*/*v*) ethanol for 24 h, the samples were washed again with PBS and then incubated with RNase A (100 *μ*g/mL) for 30 min. The samples were stained with PI (final concentration 50 *μ*g/mL) for 30 min, and flow cytometry analysis was conducted to determine the distribution of the cell cycle.

### 2.10. *In Vivo* Tumorigenesis

Male BALB/C nude mice (6 weeks of age) were purchased from Hunan SJA Laboratory Animal Co., Ltd. (Changsha, China) and housed in the animal facility at the Second Xiangya Hospital. All experimental protocols were approved by the Institutional Animal Ethics Committee at the Second Xiangya Hospital of Central South University. For tumor formation, 2 × 10^6^ MNNG/HOS cells were suspended in 200 *μ*L PBS and injected into the subcutaneous area. Lentiviral vectors of HCG9 and miR-34b-3p were constructed by GeneChem (Shanghai, China) and were administered via lateral tail vein injection before tumor cells were injected. Tumor volume was measured with a vernier caliper on days 4, 7, 11, 14, 17, 21, 24, and 28. At 28 days postsurgery, the mice were euthanized and the tumors were dissected for weight measurement.

### 2.11. Ki67 Staining

Tumor samples were harvested and fixed in 10% formalin overnight at room temperature. Samples were gradually dehydrated in ethanol and embedded in paraffin blocks. Sections of 5 *μ*m were collected, deparaffinized, and rehydrated. The antigens were retrieved by boiling in a Tris buffer (pH = 6) for 15 min using a steamer. Samples were then blocked in 1% bovine serum albumin solution (Sigma-Aldrich) and stained with the primary Ki67 antibody (rabbit, 1 : 500; ab15580; Abcam) overnight at 4°C. After washing with PBS, the samples were stained with the horseradish peroxidase (HRP) (goat; cat# ab6721; Abcam, UK) secondary antibody at 1 : 250 concentration for 1 hour at room temperature. Stained samples were mounted and observed under a Zeiss upright microscope.

### 2.12. Statistical Analysis

All data were presented as mean ± standard deviation, and all experiments were repeated independently at least 3 times. Student's *t*-test and one-way ANOVA were used to compare data between two groups and among three groups, respectively, using GraphPad Prism 8.0. *P* < 0.05 was considered statistically significant.

## 3. Results

### 3.1. HCG9 Gene Expression Was Elevated in Osteosarcoma Tissues and Cells

**Figure 1:**
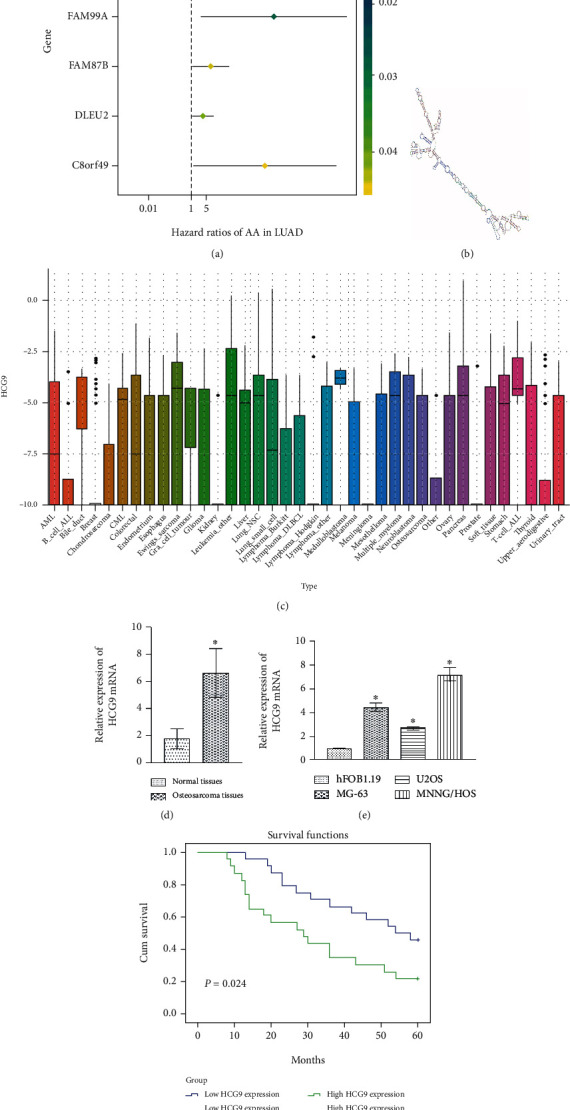
HCG9 gene expression was upregulated in osteosarcoma tissues and cells. (a) lncRNA chip data was analyzed using Cox proportional hazards regression based on the GSE21257 database, and 6 genes expressed differently in osteosarcoma tissues were screened. (b) Secondary structure prediction of HCG9 using AnnoLnc Annotation. (c) Analysis of HCG9 gene expression in the CCLE database. (d) HCG9 gene expression in osteosarcoma tumor tissue and paracarcinoma tissue. ^∗^*P* < 0.05 vs. the paracarcinoma tissues. (e) HCG9 gene expression in osteoblast and osteosarcoma cell lines. ^∗^*P* < 0.05 vs. the hFOB1.19 group. (f) Kaplan-Meier analysis of the associations between the HCG9 expression level and the overall survival of patients with osteosarcoma.

We analyzed lncRNA chip data and assessed the correlation with osteosarcoma for each lncRNA using Cox proportional hazards regression. Six genes presented statistically significant expressions (*P* < 0.05) in osteosarcoma tissues versus paracarcinoma tissues and were screened, which were MALAT1, HCG9, FAM99A, FAM87B, DLEU2, and C8orf49 (Figure 1(a)). Secondary structural identification was performed using AnnoLnc Annotation, and HCG9 was determined as our target for this study (Figure 1(b)). We analyzed HCG9 gene expression in the Cancer Cell Line Encyclopedia (CCLE) database and found that HCG9 gene expression was elevated in various cancer cell lines (Figure 1(c)). Therefore, we further validated HCG9 gene expression in osteosarcoma and paracarcinoma tissues collected from patients. The qRT-PCR result demonstrated that HCG9 gene expression was significantly upregulated in osteosarcoma tumor tissues as compared to paracarcinoma tissues (Figure 1(d)). HCG9 gene expression was also assessed in human fetal osteoblast cell line hFOB1.19 and osteosarcoma cell lines U2OS, MG-63, and MNNG/HOS, and the expression was enhanced in all three osteosarcoma cell lines as compared to hFOB1.19 cells. MNNG/HOS demonstrated the highest HCG9 gene expression. Thus, MNNG/HOS cells were selected for the following experiments in this study (Figure 1(e)). We analyzed the overall survival rate by plotting Kaplan-Meier survival plots and log-rank tests for median dichotomized HCG9 gene expression, and *P* < 0.05 was considered statistically significant. A receiver operating characteristic curve (ROC) was plotted, and the area under the curve (AUC) was analyzed to validate the association between HCG9 gene expression and prognosis (Supplemental Fig. 1). AUC at 2 years had an accuracy of 0.72. The Kaplan-Meier plot showed that the overall survival rate was significantly lower (*P* = 0.024) in the HCG9 high expression group compared with the low expression group (Figure 1(f)), suggesting HCG9 was associated with the prognosis of osteosarcoma patients.

### 3.2. HCG9 Mediated the Osteosarcoma Cell Proliferation and Apoptosis

**Figure 2:**
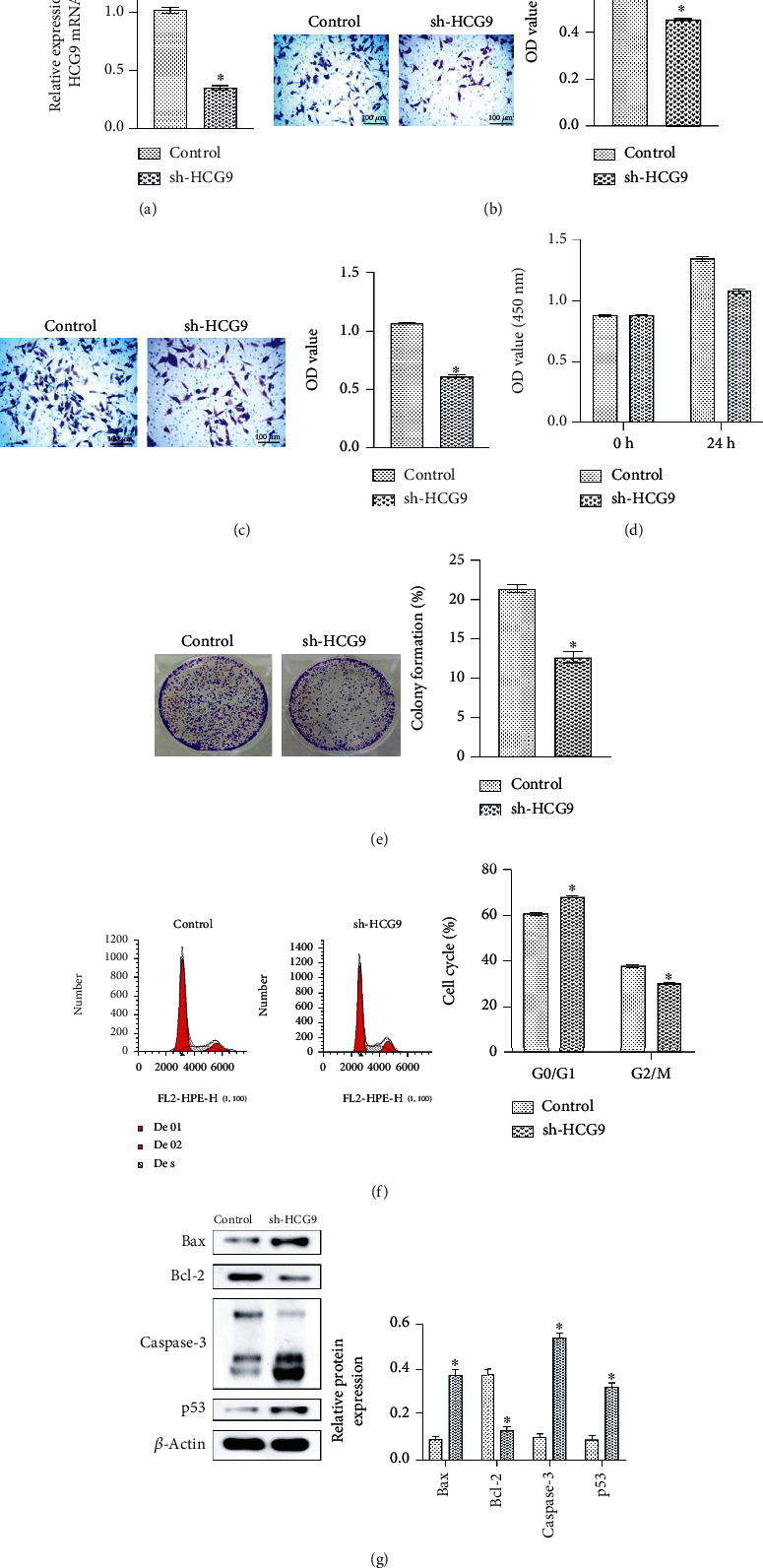
Knockdown of HCG9 inhibited MNNG/HOS cell proliferation, migration, and invasion. (a) HCG9 gene expression in the wild-type control and sh-HCG9 groups. (b) Representative images and OD value of invasion in the control and sh-HCG9 cells assessed by Transwell assay. Scale bar: 100 *μ*m. (c) Representative images and OD value of migration in the control and sh-HCG9 cells assessed by Transwell assay. Scale bar: 100 *μ*m. (d) CCK-8 assay of cell proliferation in the control and sh-HCG9 groups. (e) Representative images and cell number of colonies formed by the control and sh-HCG9 cells. (f) Cell cycle analysis by flow cytometry. (g) Apoptosis-related protein expressions (Bax, Bcl-2, Caspase-3, and p53) in the control and sh-HCG9 groups. *n* = 6 in each group. ^∗^*P* < 0.05 vs. the control group.

We next investigated how HCG9 expression impacted the proliferation and apoptosis of osteosarcoma cells. We established an HCG9 gene knockdown colony (sh-HCG9) and verified the gene expression of HCG9 with qRT-PCR. The HCG9 gene expression was significantly inhibited in the sh-HCG9 cells as compared to the wild-type control cells (Figure 2(a)). sh-HCG9 impaired the invasion and migration ability of MNNG/HOS cells. The number of invasion and migration cells was significantly lower in the sh-HCG9 group as compared to the control group (Figures 2(b) and 2(c)). Knockdown of HCG9 also suppressed MNNG/HOS cell proliferation and colony formation, as the CCK-8 assay result and colony formation result demonstrated significantly lower proliferation rates and colony counts in the sh-HCG9 group as compared to the control group (Figures 2(d) and 2(e)). sh-HCG9 induced cell arrest, as shown in the cell cycle analysis, the G0/G1 phase was significantly enhanced, and the G2/M phase was significantly inhibited in the sh-HCG9 group (Figure 2(f)). sh-HCG9 also promoted proapoptotic protein expression, including Bax, Caspase-3, and p53, and inhibited antiapoptotic protein Bcl-2 (Figure 2(g)). These results suggested that depletion of HCG9 significantly inhibited proliferation and invasion, induced cell arrest, and promoted the apoptotic phenotype of osteosarcoma cells.

### 3.3. HCG9 Expression Was Associated with Pathological Features of Osteosarcoma Patients

We analyzed the expression matrix of HCG9 and screened HCG9-associated gene expressions (*P* < 0.05). Using KEGG pathway analysis, we discovered that the high expression of HCG9 was correlated with the metastasis of osteosarcoma (Supplemental Fig. 2A). The association between HCG9 and clinicopathological features such as age, gender, metastasis, grade, and pathology state is presented in Supplemental Fig. 2B – 2F. High HCG9 expression was associated with cancer metastasis (Supplemental Fig. 2D) and grade (Supplemental Fig. 2E) but not the other pathological features such as age (Supplemental Fig. 2B) and gender (Supplemental Fig. 2C). We collected 44 osteosarcoma cases from the Second Xiangya Hospital and validated the correlation between HCG9 and clinicopathological features. Pearson's chi-squared test was performed to evaluate the *P* value. A significant association was identified between HCG9 gene expression and distant metastasis (*P* = 0.009) (Supplemental Fig. 2D) and clinical stage (*P* = 0.028) (Supplemental Fig. 2E). These results demonstrated that HCG9 gene expression was essential in osteosarcoma metastasis and progression clinically.

### 3.4. HCG9 Negatively Regulated miR-34b-3p to Mediate the Osteosarcoma Cell Proliferation and Apoptosis

**Figure 3:**
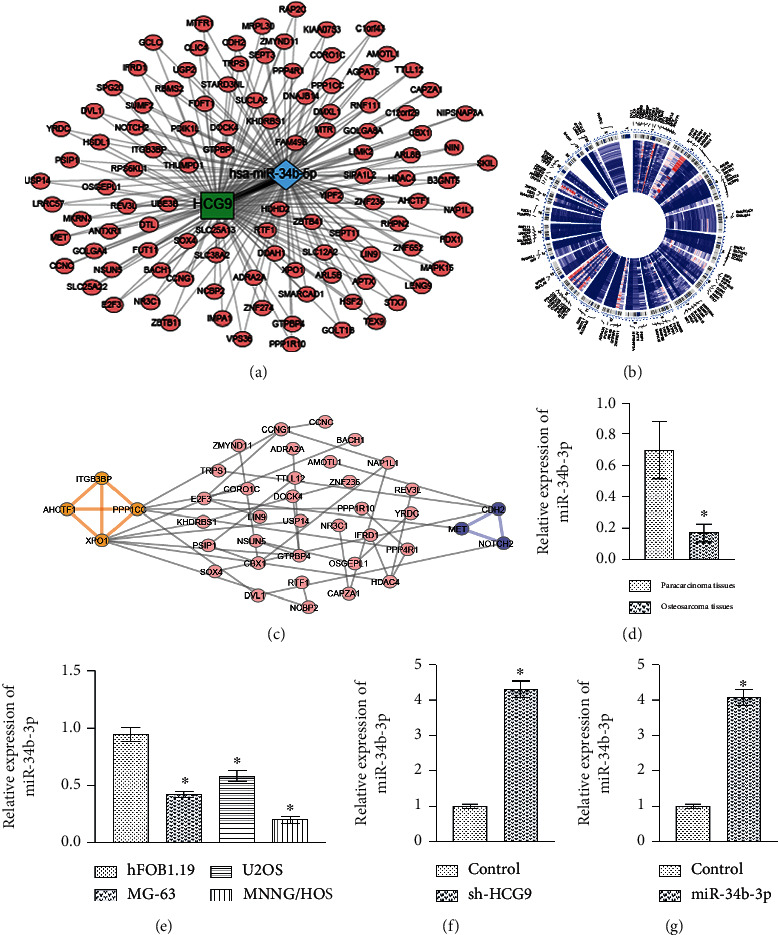
HCG9 negatively regulated miR-34b-3p. (a) starBase bioinformatic analysis predicted potential interaction between HCG9 and miR-34b-3p and mRNAs regulated by miR-34b-3p. (b) Cytoscape analysis visualized miR-34b-3p-regulated mRNA distribution in chromosomes. (c) STRING and MCODE analyses identified PPI and two different modules. (d) miR-34b-3p gene expression in osteosarcoma and paracarcinoma tissues evaluated by qRT-PCR. ^∗^*P* < 0.05 vs. the paracarcinoma tissues. (e) miR-34b-3p gene expression in osteosarcoma and osteoblast cell lines evaluated by qRT-PCR. ^∗^*P* < 0.05 vs. the hFOB1.19 group. (f) miR-34b-3p gene expressions in sh-HCG9 and control osteosarcoma cells evaluated by qRT-PCR. (g) miR-34b-3p gene expressions in miR-34b-3p and control osteosarcoma cells evaluated by qRT-PCR. *n* = 6 in each group. ^∗^*P* < 0.05 vs. the control group.

We used starBase V2.0 to predict the potential targets of HCG9, and miR-34b-3p was identified as the only target for HCG9. A total number of 104 mRNAs were pinpointed as potential targets for miR-34b-3p; thus, the ceRNA network is established and presented in Figure 3(a). The distribution of targeting mRNAs in the chromosomes was visualized by Cytoscape (Figure 3(b)). The STRING analysis (http://string-db.org/) was performed on miRNA-targeted genes, and the PPI network was constructed with 40 nodes and 46 edges. By performing MCODE, two modules were identified (Figure 3(c)). Module 1 included AHCTF1, XPO1, ITGB3BPP, and PPP1CC, which are immune response-associated genes, and module 2 included CDH2, MET, and NOTCH2, which are associated with DNA damage response [19, 20]. Gene ontology (GO), biological process (BP), and KEGG enrichment analyses showed the upregulated genes enriched in the organelle division and cell cycle pathways and the downregulated genes enriched in the miRNA in cancer and Notch signaling pathways (Supplemental Fig. 3). Gene expression of miR-34b-3p was significantly downregulated in osteosarcoma tissues and cell lines as compared to paracarcinoma tissues and osteoblast cell lines, respectively (Figures 3(d) and 3(e)). Knockdown of HCG9 significantly enhanced miR-34b-3p gene expression, suggesting HCG9 negatively regulated miR-34b-3p (Figure 3(f)). Overexpression of miR-34b-3p obviously upregulated the expression level of miR-34b-3p (Figure 3(g)).

**Figure 4:**
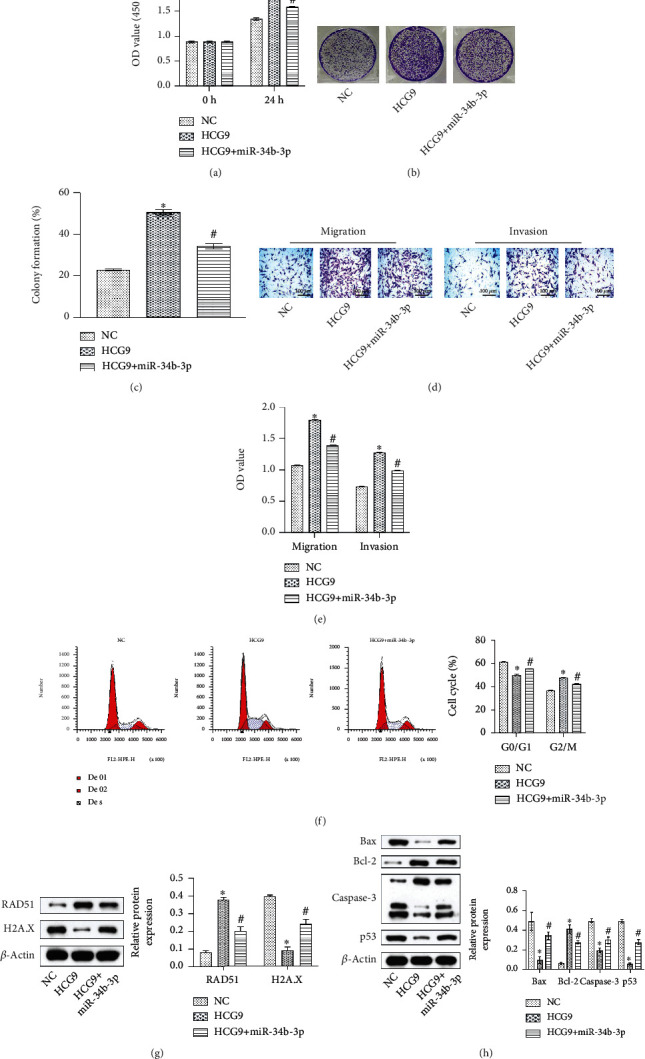
miR-34b-3p overexpression attenuated HCG9-stimulated proliferation, migration, and invasion of osteosarcoma cells. (a) CCK-8 assay of cell proliferation in the control, HCG9, and HCG9+miR-34b-3p groups. (b) Colony formation and (c) cell counts in the control, HCG9, and HCG9+miR-34b-3p groups. (d) Representative images and (e) migrated and invaded cell numbers in the control, HCG9, and HCG9+miR-34b-3p groups. (f) Cell cycle analysis of the control, HCG9, and HCG9+miR-34b-3p groups. Scale bar: 100 *μ*m. (g) Protein expression of RAD51 and H2A.X (h) Apoptosis-related protein expressions (Bax, Bcl-2, Caspase-3, and p53) in the control, HCG9, and HCG9+miR-34b-3p groups. *n* = 6 in each group. ^∗^*P* < 0.05 vs. the control group. ^#^*P* < 0.05 vs. the HCG9 group.

To verify the regulatory role of miR-34b-3p in osteosarcoma cell proliferation, migration, and invasion, we established HCG9 and miR-34b-3p overexpressing MNNG/HOS cells. HCG9 overexpression significantly enhanced cell proliferation within 24 h of culture, and cooverexpressing HCG9 and miR-34b-3p partially reversed such effect (Figure 4(a)). HCG9 promoted colony-forming ability, while cooverexpressing HCG9 and miR-34b-3p partially reduced the number of colonies as compared to the HCG9 group (Figures 4(b) and 4(c)). Cooverexpressing miR-34b-3p also inhibited MNNG/HOS cell invasion and migration that were stimulated by HCG9 overexpression (Figures 4(d) and 4(e)). The cell cycle and apoptosis-related proteins were also impacted by HCG9 and miR-34b-3p expressions. Overexpressing HCG9 encouraged cell mitosis as flow cytometry results showed that the cell number in the G2/M cycle of the HCG9 group was significantly higher than that of the control group. Cooverexpressing HCG9 and miR-34b-3p abolished such effect (Figure 4(f)). RAD51 is the key enzyme that regulates the cell cycle and preserves the G2/M phase, whereas H2A.X preserves cell cycle arrest. Overexpressing HCG9 promoted RAD51 expression and suppressed H2A.X expression, indicating HCG9 induced mitosis and hyperproliferation of the osteosarcoma cells. Cooverexpressing miR-34b-3p partially reversed such effect (Figure 4(g)). Proapoptotic proteins Bax, Caspase-3, and p53 were inhibited by HCG9 overexpression and reversed by miR-34b-3p/HCG9 cooverexpression. Antiapoptotic protein Bcl-2 was impacted in the opposite manner (Figure 4(h)). These results suggested that HCG9 enhanced proliferation and suppressed apoptosis of osteosarcoma cells by suppressing miR-34b-3p expression. Overexpressing miR-34b-3p partially reversed HCG9-stimulated hyperproliferation.

### 3.5. miR-34b-3p Induced Cell Cycle Arrest via Downregulating RAD51

**Figure 5:**
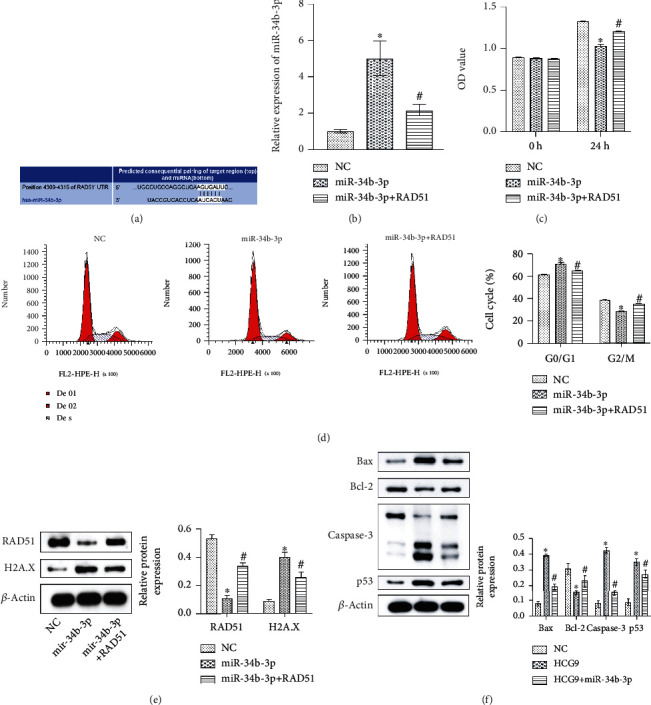
miR-34b-3p downregulated RAD51 to inhibit osteosarcoma cell proliferation. (a) Potential binding sites between miR-34b-3p and RAD51. (b) Gene expression of miR-34b-3p. (c) Cell proliferation. (d) Cell cycle analysis. (e) RAD51 and H2A.X protein expression. (f) Apoptosis-related protein expressions (Bax, Bcl-2, Caspase-3, and p53) in the control, miR-34b-3p, and miR-34b-3p+RAD51 groups. ^∗^*P* < 0.05 vs. the control group. ^#^*P* < 0.05 vs. the miR-24b-3p group.

We next evaluated the potential target of miR-34b-3p. RAD51 regulated the cell cycle and DNA damage checkpoint, and these pathways were significantly upregulated in our clinical osteosarcoma tissue samples (Supplemental Fig. 3). The potential binding site between miR-34b-3p and RAD51 was predicted using starBase (Figure 5(a)). We established miR-34b-3p overexpressing and miR-34b-3p/RAD51 cooverexpressing osteosarcoma cells to evaluate the involvement of RAD51. The expression of miR-34b-3p was significantly upregulated in the miR-34b-3p overexpression group and was partially reversed in the miR-34b-3p/RAD51 cooverexpression group (Figure 5(b)). CCK-8 assay results showed that miR-34b-3p overexpression inhibited cell proliferation as compared to the control group, whereas miR-34b-3p/RAD51 cooverexpression recovered the proliferation of osteosarcoma cells (Figure 5(c)). RAD51 also played a role in the miR-34b-3p-mediated cell cycle, and miR-34b-3p overexpression induced cell cycle arrest, as it reduced the cell number in the G2/M phase as compared to the control group, while miR-34b-3p/RAD51 cooverexpression restored the G2/M phase (Figure 5(d)). RAD51 protein expression was significantly inhibited by miR-34b-3p overexpression and restored by miR-34b-3p/RAD51 cooverexpression. Cell cycle arrest marker H2A.X was impacted oppositely (Figure 5(e)). miR-34b-3p enhanced the expressions of proapoptotic proteins, including Bax, Caspase-3, and p53, and inhibited the expression of antiapoptotic protein Bcl-2. miR-34b-3p/RAD51 cooverexpression partially reversed such effect of miR-34b-3p (Figure 5(f)). These results indicated that RAD51 could serve as the potential target of miR-34b-3p and played a critical role in the HCG9/miR-34b-3p functioning axis.

### 3.6. HCG9 Promoted Osteosarcoma Progression while miR-34b-3p Ameliorated HCG9-Stimulated Osteosarcoma Progression *In Vivo*

**Figure 6:**
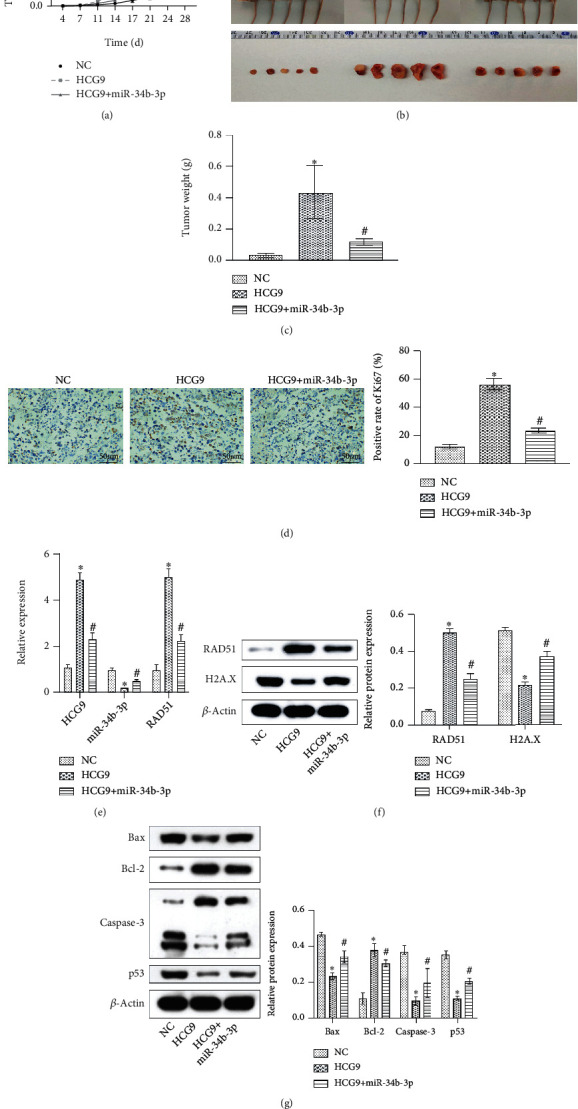
miR-34b-3p ameliorated HCG9-stimulated osteosarcoma progression *in vivo*. (a) Quantification of tumor volume. (b) Representative images of tumor growth. (c) Quantification of tumor weight in the control, HCG9, and HCG9+miR-34b-3p groups. (d) Ki67 staining of tumor tissue and the positive rate. Scale bar: 100 *μ*m. (e) Gene expression of HCG9, miR-34b-3p, and RAD51. (f) Protein expression of RAD51 and H2A.X. (g) Apoptosis-related protein expressions (Bax, Bcl-2, Caspase-3, and p53) in the control, HCG9, and HCG9+miR-34b-3p groups. ^∗^*P* < 0.05 vs. the control group. ^#^*P* < 0.05 vs. the HCG9 group.

We evaluated the role of HCG9 and miR-34b-3p in regulating osteosarcoma progression *in vivo* using a nude mouse model. As compared to the tumor control, HCG9 overexpression significantly aggregated the tumor growth, and coexpressing miR-34b-3p partially attenuated the HCG9-stimulated tumor growth, as evidenced by tumor volume and weight analysis (Figures 6(a) – 6(c)). The immunohistochemistry staining result showed excessive Ki67-positive cells in the HCG9 overexpressing group, and the HCG9/miR-34b-3p cooverexpressing group partially reduced the Ki67-positive cell number (Figure 6(d)), indicating miR-34b-3p mitigated the tumor aggressiveness stimulated by HCG9. Gene expressions of HCG9, miR-34b-3p, and RAD51 were evaluated by qRT-PCR to validate the overexpressing efficiency. HCG9 overexpression suppressed miR-34b-3p and promoted RAD51, whereas cooverexpressing HCG9/miR-34b-3p reversed such effect (Figure 6(e)). Western blot results further confirmed that the protein expression of RAD51 was positively impacted by HCG9 overexpression and reversed by HCG9/miR-34b-3p cooverexpression. H2A.X protein expression was impacted in the opposite manner (Figure 6(f)). HCG9 overexpression induced an antiapoptotic phenotype in the tumor tissue, as it suppressed proapoptotic Bax, Caspase-3, and p53 expressions and promoted antiapoptotic Bcl-2 expression. Cooverexpressing HCG9/miR-34b-3p partially blocked antiapoptotic effect (Figure 6(g)). The *in vivo* results confirmed that HCG9 aggravated tumor progression and miR-34b-3p reversed such effect.

## 4. Discussions

Distant metastases are often associated with osteosarcoma after surgery. It is reported that metastases occurred in over 85% of patients who had osteosarcoma [21], and the metastasis drastically aggravated the prognosis of osteosarcoma [22]. Recent studies have suggested that lncRNAs exhibited important regulatory functions in regulating metastasis of osteosarcoma [23]. In our current study, we developed a genome-wide approach to link lncRNA HCG9 to clinicopathological features of osteosarcoma, especially metastasis. We discovered that high HCG9 was correlated with metastasis and severe cancer grades. The proliferation, migration, and invasion of osteosarcoma cells responded to the alteration of HCG9 gene expression. Using the bioinformatic technique, we established the ceRNA network of HCG9 and the targeting miRNA was determined. To our knowledge, this is the first study that reported the essential regulatory role of HCG9 and its underlying mechanism in osteosarcoma progression.

Dysregulated lncRNA gene expression was commonly found in tumor tissues. For osteosarcoma, in particular, an increasing number of studies have reported various lncRNAs expressed differently in tumor tissues and paracarcinoma tissues [9]. RNA microarray is a powerful tool to screen abnormal lncRNA expressions in tumors and paracarcinoma tissues, providing potential candidates for gene therapy [24]. Over 25,000 lncRNAs were analyzed by microarray, and thousands of osteosarcoma-associated genes were identified [25]. Among these identified lncRNAs, the role of a small number of tumor-suppressive and oncogenic lncRNAs in osteosarcoma has been elucidated. For example, MALAT1 is one of the first identified cancer-associated genes, and it is commonly studied in osteosarcoma. Wang et al. reported that lncRNA MALAT1 was highly expressed in osteosarcoma tumor tissues and promoted tumor metastasis [26]. Other oncogenic lncRNAs such as H19, HULC, SNHG12, and HOTAIR also demonstrated their regulatory functions in promoting cancer cell proliferation, inhibiting apoptosis, and aggravating tumor progression [27–30]. Herein, we screened 6 osteosarcoma-related genes by lncRNA microarray, MALAT1, HCG9, FAM99A, FAM87B, DLEU2, and C8orf49. MALAT1 and DLEU2 have been shown to promote osteosarcoma progression in previous studies [31, 32]. FAM99A, FAM87B, and C8orf49 have not been reported in osteosarcoma studies and are rarely reported in other cancers. Due to the limitation of funds and time, we did not carry out in-depth research on them. In future studies, we will further determine whether they are associated with osteosarcoma. HCG9 has been briefly reported in breast cancer and gastric cancer [33, 34] but never in osteosarcoma. By analyzing CCLE, we found that HCG9 was associated with various cancer progressions. Further bioinformatic analysis showed that high HCG9 expression was associated with osteosarcoma progression. Therefore, we finally chose HCG9 as our research object. Additional validation was conducted by comparing HCG9 expression in clinical osteosarcoma samples with paracarcinoma tissues, and abnormally elevated HCG9 expression was confirmed in tumor samples. Knockdown of HCG9 significantly suppressed osteosarcoma cell proliferation, migration, and invasion, demonstrating an oncogenic role of HCG9. To our knowledge, HCG9 was for the first time reported to be associated with osteosarcoma progression, and our results provided a new biomarker for osteosarcoma diagnosis and treatment.

One of the main functions of lncRNAs was acting as ceRNAs to regulate miRNA expressions. ceRNAs are a novel class of posttranscriptional regulators of gene expressions, which compete for the binding to miRNA/mRNA recognition elements [35]. Establishing a ceRNA network using bioinformatic techniques facilitates elucidating the possible impacts on the major signaling pathways in cancer progression. Huang et al. analyzed the differentially expressed ceRNAs associated with recurrent soft tissue sarcomas based on The Cancer Genome Atlas (TCGA) database, providing comprehensive information for improving personalized management for soft tissue sarcoma [36]. Several identified ceRNA axes in osteosarcoma were studied in detail in *in vitro* and *in vivo* animal models. For instance, Zheng et al. reported that lncRNA SNHG3 is competitively bound to miR-151a-3p to regulate RAB22A activity and mediate osteosarcoma cell invasion and migration [18]. Xie et al. found that TUG1 could potentially bind to miR-9-5p and miR-212-3p to regulate different signaling pathways in osteosarcoma progression [37, 38], revealing the details of two small pieces in the entire puzzle of the ceRNA network. In our current study, miR-34b-3p was predicted by starBase as the only potential target of HCG9. The miR-34 family is commonly downregulated in different cancers [39], including ovarian cancer and pancreatic cancer [40, 41]. However, there has been no report of miR-34b-3p in regulating osteosarcoma progression. Herein, we established the ceRNA network that linked HCG9 with miR-34b-3p, and the signaling pathways impacted by these ncRNAs were also elucidated. The ceRNA network and pathway analysis pinpointed 4 DNA damage-related pathways involving the DNA damage checkpoint and mitotic cell cycle, indicating the HCG9/miR-34b-3p axis played a major role in the DNA repair and cell cycle. We identified RAD51, the key enzyme that promoted mitosis, as the potential target of miR-34b-3p and found that miR-34b-3p induced cell cycle arrest by downregulating RAD51, and HCG9 negatively regulated miR-34b-3p. Therefore, positively regulated RAD51 promoted proliferation and inhibited apoptosis of osteosarcoma cells. We did not perform the dual-luciferase report experiment due to time and investigation funds. It is indeed necessary to verify the relationship between miR-34b-3p and RAD51 through the dual-luciferase report experiment. We have planned this verification in future studies.

In summary, our data demonstrated that HCG9 negatively regulated miR-34b-3p, and abnormally low expressions of miR-34b-3p in osteosarcoma tumor tissues supported our hypothesis. By overexpressing miR-34b-3p, the *in vitro* hyperproliferation, migration, and invasion, as well as *in vivo* tumor growth stimulated by HCG9 overexpression, were mitigated, suggesting a potential protective role of miR-34b-3p in osteosarcoma progression.

## 5. Conclusion

HCG9 gene expression increased in osteosarcoma tissues and cell lines, and high HCG9 expression was particularly associated with osteosarcoma metastasis. miR-34b-3p served as the target of HCG9, and RAD51 was the potential target of miR-34b-3p. Overexpressing HCG9 promoted osteosarcoma cell proliferation, migration, and invasion *in vitro*, as well as aggravated tumor progression *in vivo*. Cooverexpression of miR-34b-3p and HCG9 was able to attenuate the osteosarcoma progression. Our study provided novel therapeutic targets that will assist osteosarcoma treatment in clinical practice.

## Figures and Tables

**Table 1:** Primer sequences.

Gene	Sequences (5′-3′)
HCG9	F: AGTCAGGAGCCCCATAGTCCC
	R: CCGCTGAGGCAGTCACATCCC

miR-34b-5p	F: CCAATCACTAACTCCACTGCCAT

RAD51	F: GAGCGTTCAACACAGACCAC
	R: AGTGCATACCTAGATTCTACCAT

*β*-Actin	F: ACCCTGAAGTACCCCATCGAG
	R: AGCACAGCCTGGATAGCAAC

U6	F: CTCGCTTCGGCAGCACA
	R: AACGCTTCACGAATTTGCGT

## Data Availability

The authors confirm that all data underlying the findings are available. All relevant data are within the paper and its supporting information files.

## Supplementary Materials

Supplementary Materials Supplemental Fig. 1 (A) ROC plot and (B) AUC analysis of the correlation between HCG9 gene expression and survival rates. Supplemental Fig. 2 (A) Heat map of HCG9 expression and gene expressions. Correlation between HCG9 expression and (B) age, (C) gender, (D) metastasis, (E) grade, and (F) pathology state. Supplemental Fig. 3 GO, BP, and KEGG analyses of the enrichment of mRNAs regulated by the HCG9/miR-34b-3p axis in signaling pathways.
