# Supplementary figures and images for: Long Noncoding RNA HCG9 Promotes Osteosarcoma Progression through RAD51 by Acting as a ceRNA of miR-34b-3p
Source: Mediators Inflamm. 2021 Aug 18;2021:9978882. doi: 10.1155/2021/9978882 (PMC8390166; doi:10.1155/2021/9978882)

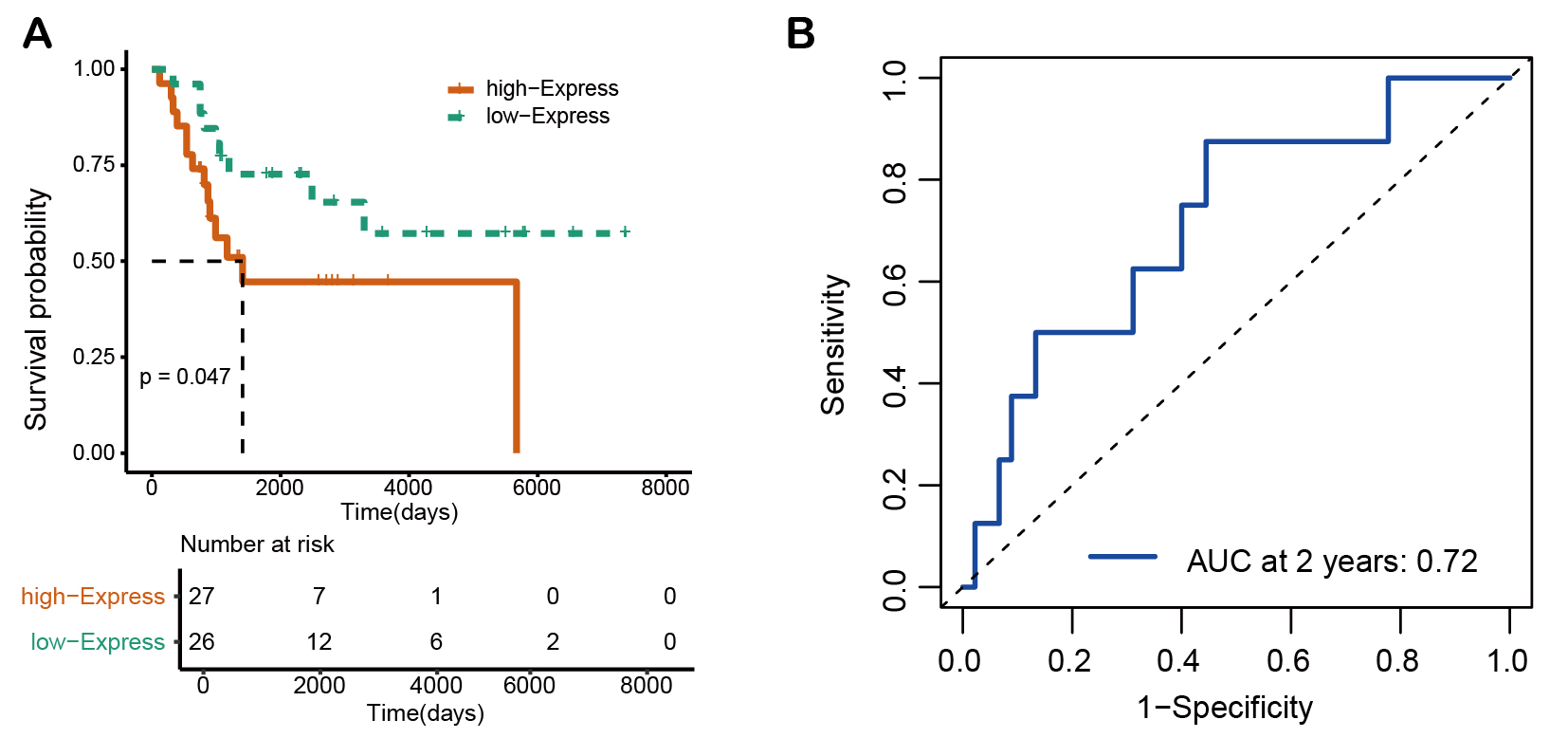

Supplement: Supplementary Materials — Supplemental Fig. 1 (A) ROC plot and (B) AUC analysis of the correlation between HCG9 gene expression and survival rates. Supplemental Fig. 2 (A) Heat map of HCG9 expression and gene expressions. Correlation between HCG9 expression and (B) age, (C) gender, (D) metastasis, (E) grade, and (F) pathology state. Supplemental Fig. 3 GO, BP, and KEGG analyses of the enrichment of mRNAs regulated by the HCG9/miR-34b-3p axis in signaling pathways. [file 9978882.f1.zip › 9978882.f1/S1 (1).jpg]

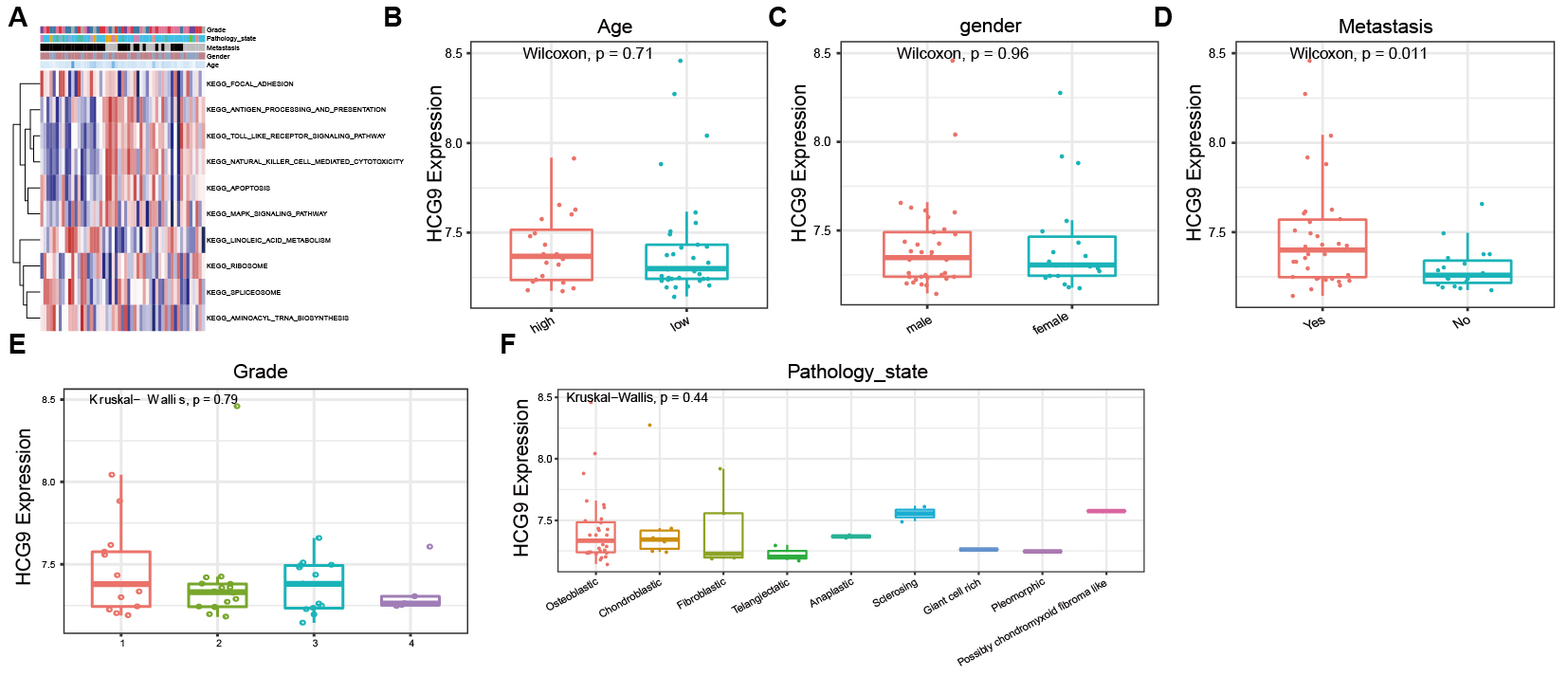

Supplement: Supplementary Materials — Supplemental Fig. 1 (A) ROC plot and (B) AUC analysis of the correlation between HCG9 gene expression and survival rates. Supplemental Fig. 2 (A) Heat map of HCG9 expression and gene expressions. Correlation between HCG9 expression and (B) age, (C) gender, (D) metastasis, (E) grade, and (F) pathology state. Supplemental Fig. 3 GO, BP, and KEGG analyses of the enrichment of mRNAs regulated by the HCG9/miR-34b-3p axis in signaling pathways. [file 9978882.f1.zip › 9978882.f1/S2 (1).jpg]

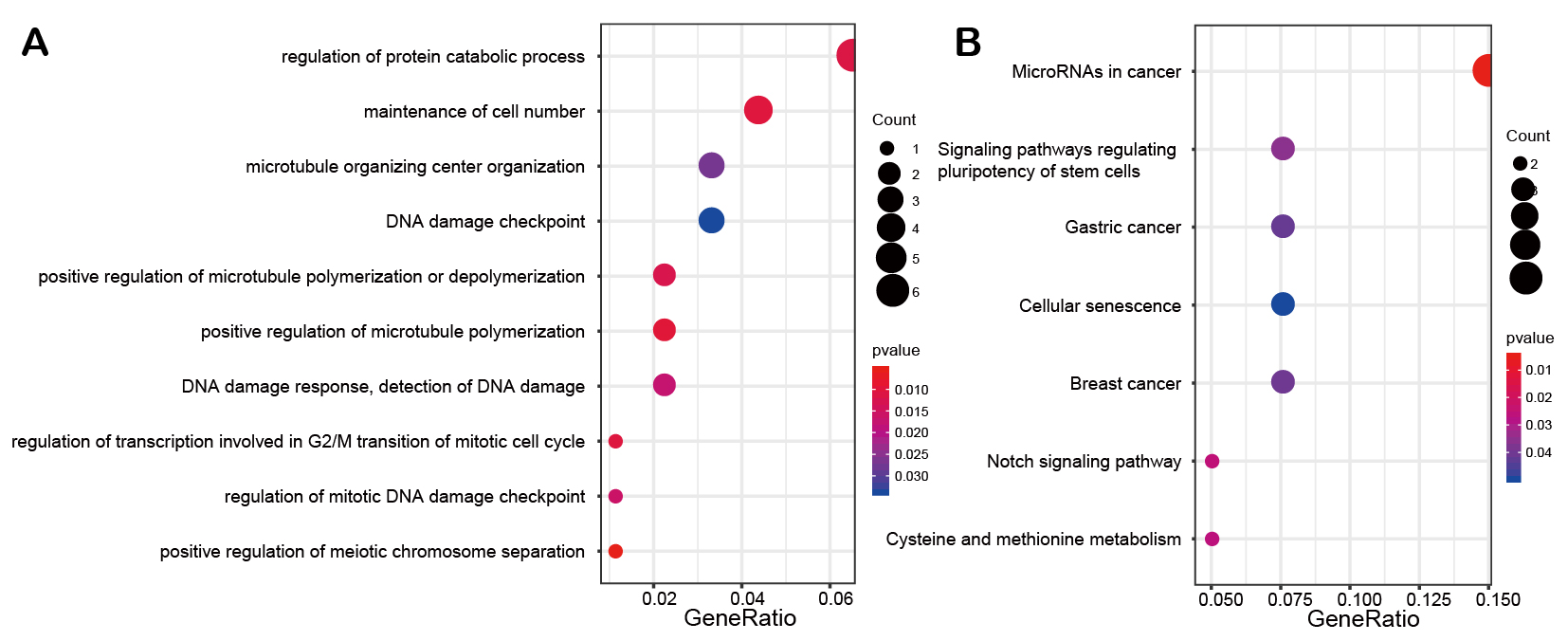

Supplement: Supplementary Materials — Supplemental Fig. 1 (A) ROC plot and (B) AUC analysis of the correlation between HCG9 gene expression and survival rates. Supplemental Fig. 2 (A) Heat map of HCG9 expression and gene expressions. Correlation between HCG9 expression and (B) age, (C) gender, (D) metastasis, (E) grade, and (F) pathology state. Supplemental Fig. 3 GO, BP, and KEGG analyses of the enrichment of mRNAs regulated by the HCG9/miR-34b-3p axis in signaling pathways. [file 9978882.f1.zip › 9978882.f1/S3 (1).jpg]
